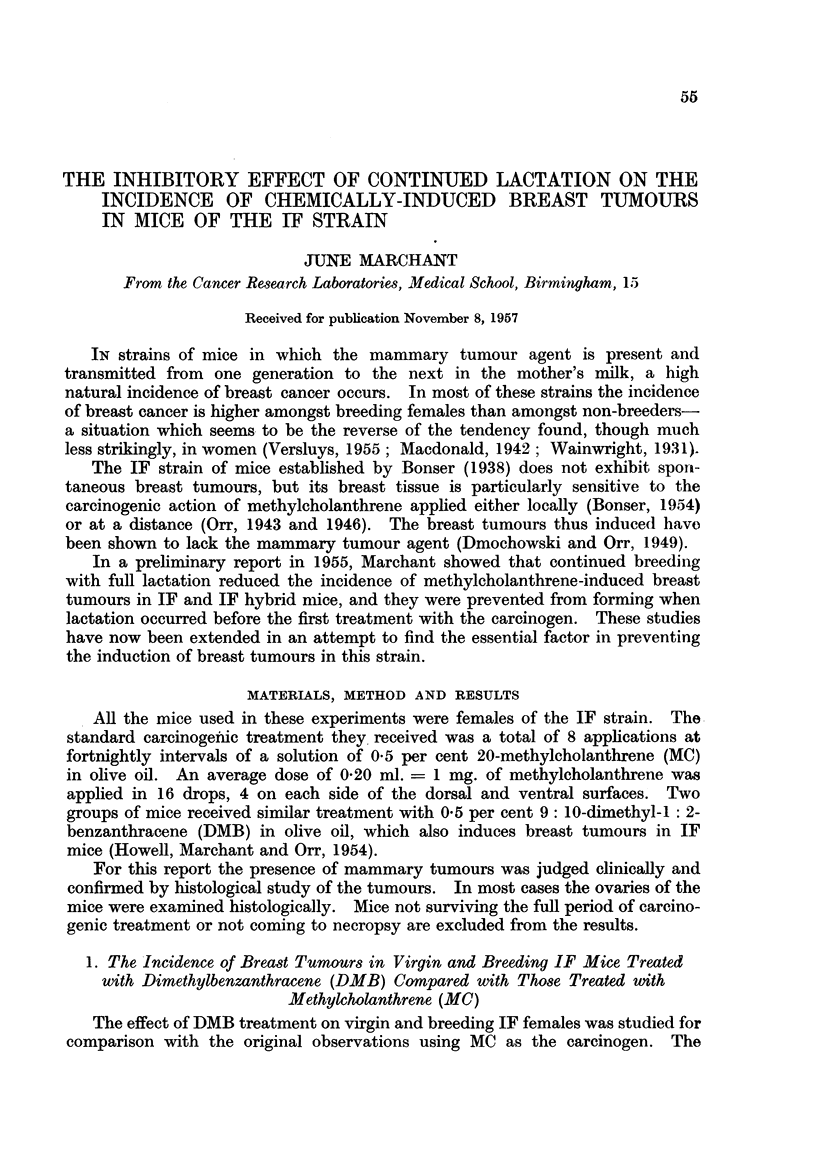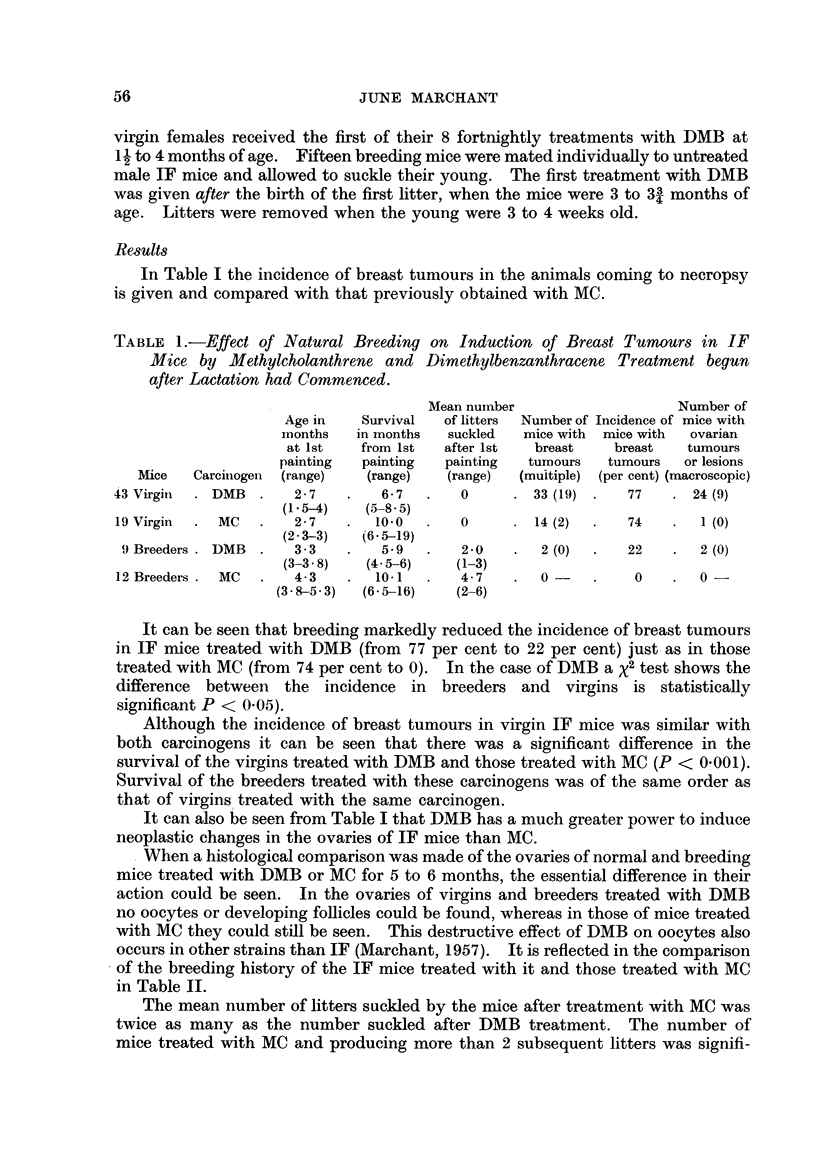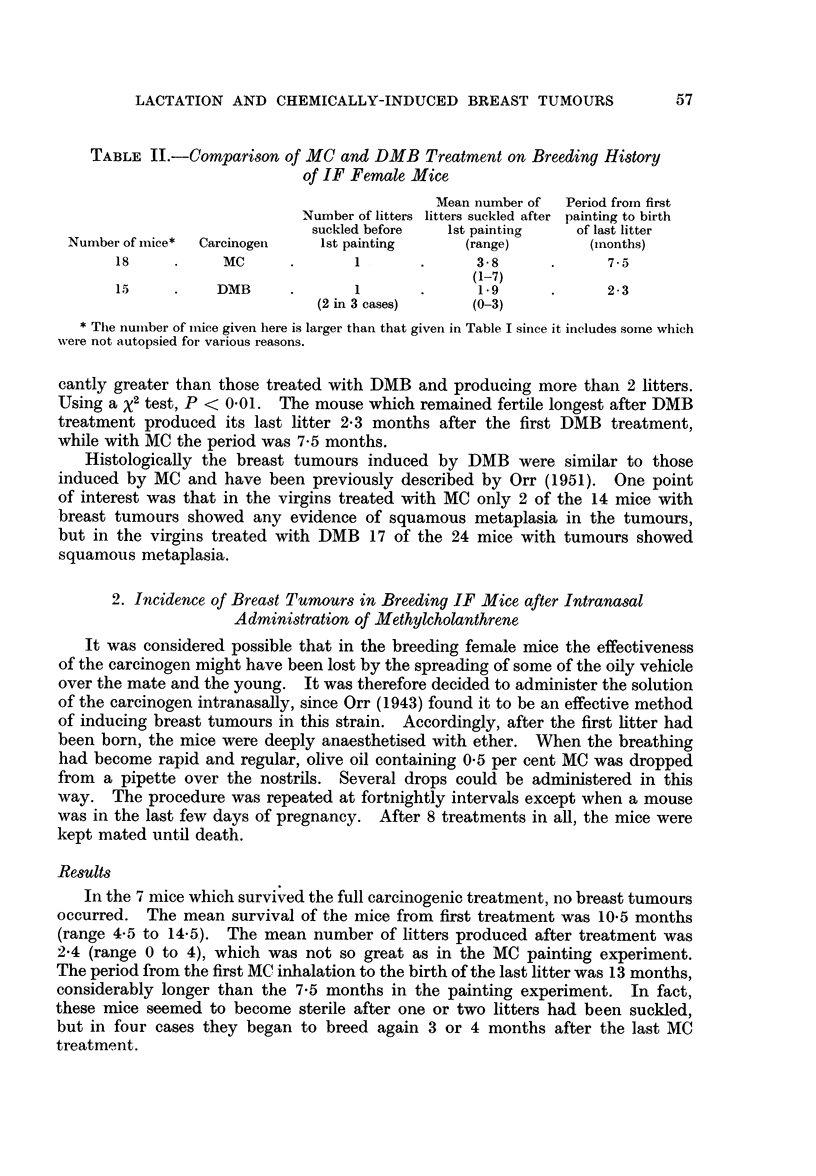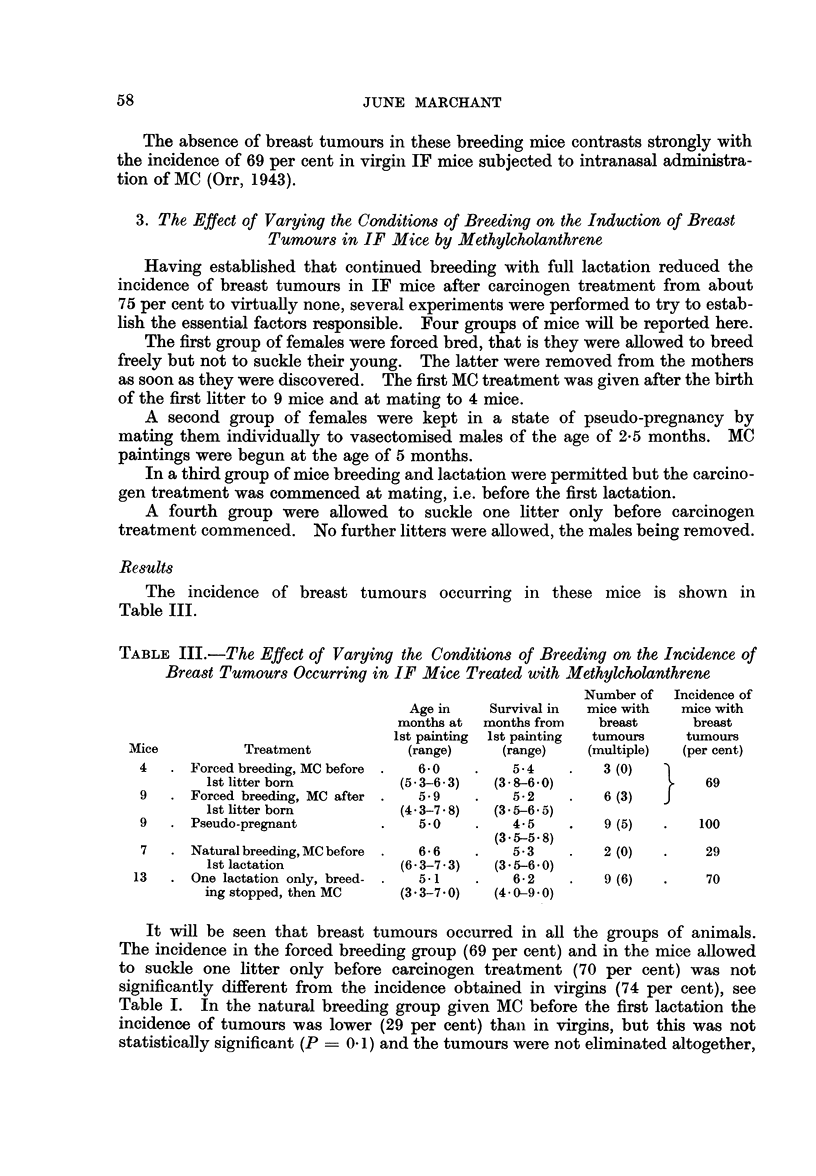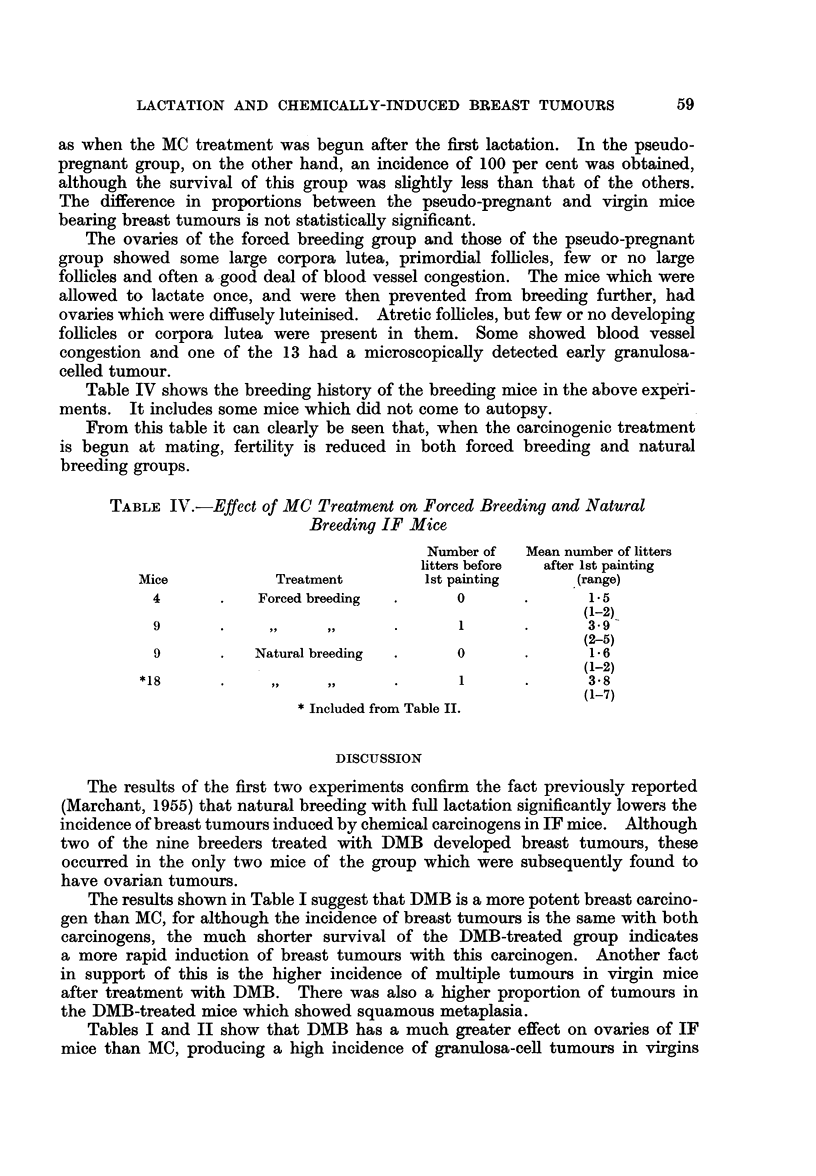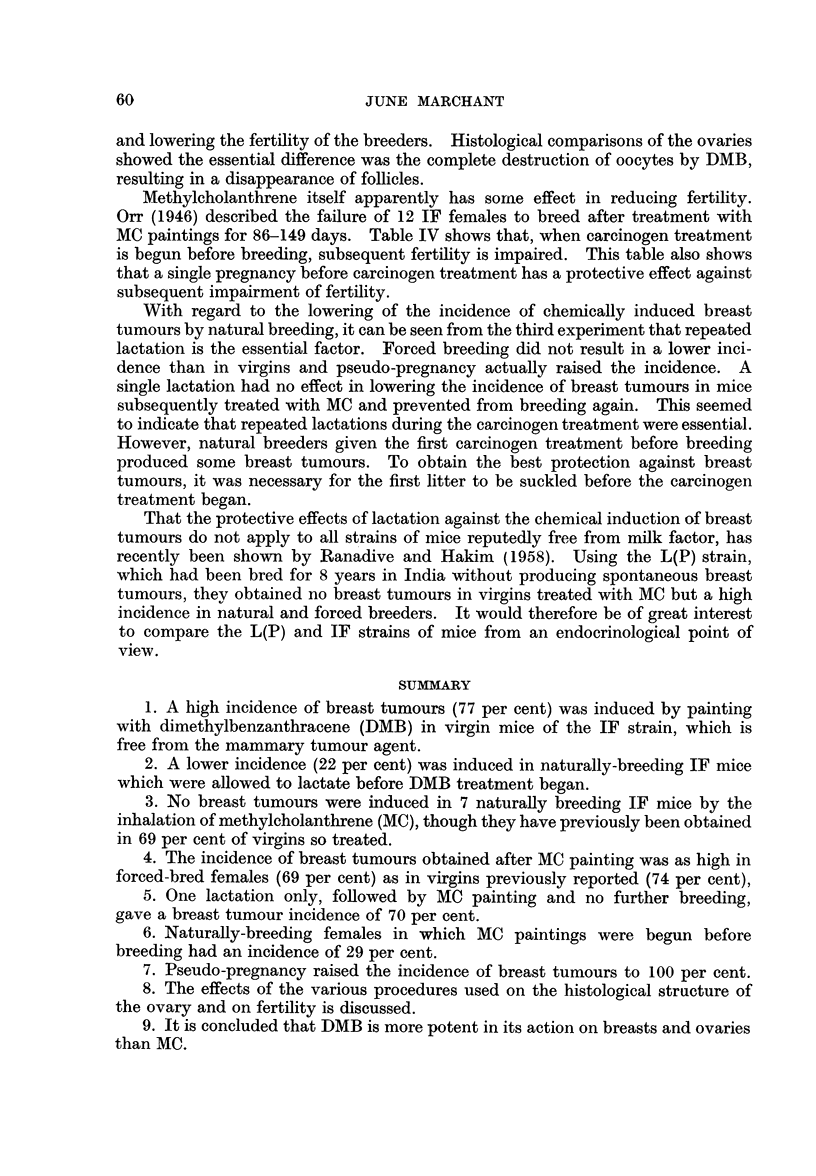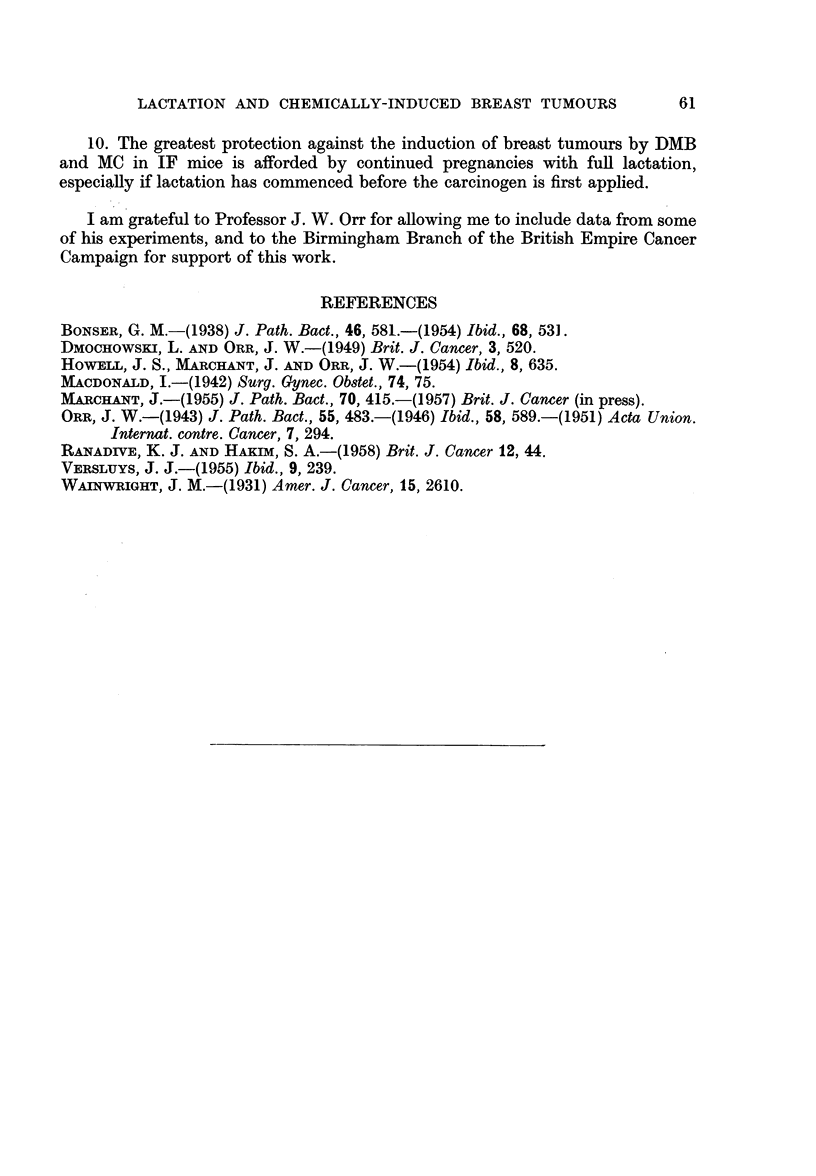# The Inhibitory Effect of Continued Lactation on the Incidence of Chemically-Induced Breast Tumours in Mice of the IF Strain

**DOI:** 10.1038/bjc.1958.8

**Published:** 1958-03

**Authors:** June Marchant


### THE INHIBITORY EFFECT OF CONTINUED LACTATION ON THE INCIDENCE OF CHEMICALLY-INDUCED BREAST TUMOURS IN MICE OF THE IF STRAIN

JUNE MARCHANT

*From the Cancer Research Laboratories, Medical School, Birmingham,* 15

Received for publication November 8, 1957

In strains of mice in which the mammary tumour agent is present and transmitted from one generation to the next in the mother's milk, a high natural incidence of breast cancer occurs. In most of these strains the incidence of breast cancer is higher amongst breeding females than amongst non-breeders—a situation which seems to be the reverse of the tendency found, though much less strikingly, in women (Versluys, 1955 ; Macdonald, 1942 ; Wainwright, 1931).

The IF strain of mice established by Bonser (1938) does not exhibit spontaneous breast tumours, but its breast tissue is particularly sensitive to the carcinogenic action of methylcholanthrene applied either locally (Bonser, 1954) or at a distance (Orr, 1943 and 1946). The breast tumours thus induced have been shown to lack the mammary tumour agent (Dmochowski and Orr, 1949).

In a preliminary report in 1955, Marchant showed that continued breeding with full lactation reduced the incidence of methylcholanthrene-induced breast tumours in IF and IF hybrid mice, and they were prevented from forming when lactation occurred before the first treatment with the carcinogen. These studies have now been extended in an attempt to find the essential factor in preventing the induction of breast tumours in this strain.

### MATERIALS, METHOD AND RESULTS

All the mice used in these experiments were females of the IF strain. The standard carcinogenic treatment they received was a total of 8 applications at fortnightly intervals of a solution of 0·5 per cent 20-methylcholanthrene (MC) in olive oil. An average dose of 0·20 ml. = 1 mg. of methylcholanthrene was applied in 16 drops, 4 on each side of the dorsal and ventral surfaces. Two groups of mice received similar treatment with 0·5 per cent 9 : 10-dimethyl-1 : 2-benzanthracene (DMB) in olive oil, which also induces breast tumours in IF mice (Howell, Marchant and Orr, 1954).

For this report the presence of mammary tumours was judged clinically and confirmed by histological study of the tumours. In most cases the ovaries of the mice were examined histologically. Mice not surviving the full period of carcinogenic treatment or not coming to necropsy are excluded from the results.

### 1. *The Incidence of Breast Tumours in Virgin and Breeding IF Mice Treated with Dimethylbenzanthracene (DMB) Compared with Those Treated with Methylcholanthrene (MC)*

The effect of DMB treatment on virgin and breeding IF females was studied for comparison with the original observations using MC as the carcinogen. The virgin females received the first of their 8 fortnightly treatments with DMB at $1\frac{1}{2}$ to 4 months of age. Fifteen breeding mice were mated individually to untreated male IF mice and allowed to suckle their young. The first treatment with DMB was given *after* the birth of the first litter, when the mice were 3 to $3\frac{3}{4}$ months of age. Litters were removed when the young were 3 to 4 weeks old.

*Results*

In Table I the incidence of breast tumours in the animals coming to necropsy is given and compared with that previously obtained with MC.

TABLE I.—*Effect of Natural Breeding on Induction of Breast Tumours in IF Mice by Methylcholanthrene and Dimethylbenzanthracene Treatment begun after Lactation had Commenced.*

| Mice | Carcinogen | Age in months at 1st painting (range) | Survival in months from 1st painting (range) | Mean number of litters suckled after 1st painting (range) | Number of mice with breast tumours (multiple) | Incidence of mice with breast tumours (per cent) | Number of mice with ovarian tumours or lesions (macroscopic) |
|---|---|---|---|---|---|---|---|
| 43 Virgin | DMB | 2·7 (1·5–4) | 6·7 (5–8·5) | 0 | 33 (19) | 77 | 24 (9) |
| 19 Virgin | MC | 2·7 (2·3–3) | 10·0 (6·5–19) | 0 | 14 (2) | 74 | 1 (0) |
| 9 Breeders | DMB | 3·3 (3–3·8) | 5·9 (4·5–6) | 2·0 (1–3) | 2 (0) | 22 | 2 (0) |
| 12 Breeders | MC | 4·3 (3·8–5·3) | 10·1 (6·5–16) | 4·7 (2–6) | 0 — | 0 | 0 — |

It can be seen that breeding markedly reduced the incidence of breast tumours in IF mice treated with DMB (from 77 per cent to 22 per cent) just as in those treated with MC (from 74 per cent to 0). In the case of DMB a $\chi^2$ test shows the difference between the incidence in breeders and virgins is statistically significant $P < 0{\cdot}05$).

Although the incidence of breast tumours in virgin IF mice was similar with both carcinogens it can be seen that there was a significant difference in the survival of the virgins treated with DMB and those treated with MC ($P < 0{\cdot}001$). Survival of the breeders treated with these carcinogens was of the same order as that of virgins treated with the same carcinogen.

It can also be seen from Table I that DMB has a much greater power to induce neoplastic changes in the ovaries of IF mice than MC.

When a histological comparison was made of the ovaries of normal and breeding mice treated with DMB or MC for 5 to 6 months, the essential difference in their action could be seen. In the ovaries of virgins and breeders treated with DMB no oocytes or developing follicles could be found, whereas in those of mice treated with MC they could still be seen. This destructive effect of DMB on oocytes also occurs in other strains than IF (Marchant, 1957). It is reflected in the comparison of the breeding history of the IF mice treated with it and those treated with MC in Table II.

The mean number of litters suckled by the mice after treatment with MC was twice as many as the number suckled after DMB treatment. The number of mice treated with MC and producing more than 2 subsequent litters was signifi-

TABLE II.—*Comparison of MC and DMB Treatment on Breeding History of IF Female Mice*

| Number of mice* | Carcinogen | Number of litters suckled before 1st painting | Mean number of litters suckled after 1st painting (range) | Period from first painting to birth of last litter (months) |
|---|---|---|---|---|
| 18 | MC | 1 | 3·8 (1–7) | 7·5 |
| 15 | DMB | 1 (2 in 3 cases) | 1·9 (0–3) | 2·3 |

* The number of mice given here is larger than that given in Table I since it includes some which were not autopsied for various reasons.

cantly greater than those treated with DMB and producing more than 2 litters. Using a $\chi^2$ test, $P < 0{\cdot}01$. The mouse which remained fertile longest after DMB treatment produced its last litter 2·3 months after the first DMB treatment, while with MC the period was 7·5 months.

Histologically the breast tumours induced by DMB were similar to those induced by MC and have been previously described by Orr (1951). One point of interest was that in the virgins treated with MC only 2 of the 14 mice with breast tumours showed any evidence of squamous metaplasia in the tumours, but in the virgins treated with DMB 17 of the 24 mice with tumours showed squamous metaplasia.

### 2. *Incidence of Breast Tumours in Breeding IF Mice after Intranasal Administration of Methylcholanthrene*

It was considered possible that in the breeding female mice the effectiveness of the carcinogen might have been lost by the spreading of some of the oily vehicle over the mate and the young. It was therefore decided to administer the solution of the carcinogen intranasally, since Orr (1943) found it to be an effective method of inducing breast tumours in this strain. Accordingly, after the first litter had been born, the mice were deeply anaesthetised with ether. When the breathing had become rapid and regular, olive oil containing 0·5 per cent MC was dropped from a pipette over the nostrils. Several drops could be administered in this way. The procedure was repeated at fortnightly intervals except when a mouse was in the last few days of pregnancy. After 8 treatments in all, the mice were kept mated until death.

### *Results*

In the 7 mice which survived the full carcinogenic treatment, no breast tumours occurred. The mean survival of the mice from first treatment was 10·5 months (range 4·5 to 14·5). The mean number of litters produced after treatment was 2·4 (range 0 to 4), which was not so great as in the MC painting experiment. The period from the first MC inhalation to the birth of the last litter was 13 months, considerably longer than the 7·5 months in the painting experiment. In fact, these mice seemed to become sterile after one or two litters had been suckled, but in four cases they began to breed again 3 or 4 months after the last MC treatment.

The absence of breast tumours in these breeding mice contrasts strongly with the incidence of 69 per cent in virgin IF mice subjected to intranasal administration of MC (Orr, 1943).

### 3. *The Effect of Varying the Conditions of Breeding on the Induction of Breast Tumours in IF Mice by Methylcholanthrene*

Having established that continued breeding with full lactation reduced the incidence of breast tumours in IF mice after carcinogen treatment from about 75 per cent to virtually none, several experiments were performed to try to establish the essential factors responsible. Four groups of mice will be reported here.

The first group of females were forced bred, that is they were allowed to breed freely but not to suckle their young. The latter were removed from the mothers as soon as they were discovered. The first MC treatment was given after the birth of the first litter to 9 mice and at mating to 4 mice.

A second group of females were kept in a state of pseudo-pregnancy by mating them individually to vasectomised males of the age of 2·5 months. MC paintings were begun at the age of 5 months.

In a third group of mice breeding and lactation were permitted but the carcinogen treatment was commenced at mating, i.e. before the first lactation.

A fourth group were allowed to suckle one litter only before carcinogen treatment commenced. No further litters were allowed, the males being removed.

*Results*

The incidence of breast tumours occurring in these mice is shown in Table III.

TABLE III.—*The Effect of Varying the Conditions of Breeding on the Incidence of Breast Tumours Occurring in IF Mice Treated with Methylcholanthrene*

| Mice | Treatment | Age in months at 1st painting (range) | Survival in months from 1st painting (range) | Number of mice with breast tumours (multiple) | Incidence of mice with breast tumours (per cent) |
|---|---|---|---|---|---|
| 4 | Forced breeding, MC before 1st litter born | 6·0 (5·3–6·3) | 5·4 (3·8–6·0) | 3 (0) | 69 |
| 9 | Forced breeding, MC after 1st litter born | 5·9 (4·3–7·8) | 5·2 (3·5–6·5) | 6 (3) | |
| 9 | Pseudo-pregnant | 5·0 | 4·5 (3·5–5·8) | 9 (5) | 100 |
| 7 | Natural breeding, MC before 1st lactation | 6·6 (6·3–7·3) | 5·3 (3·5–6·0) | 2 (0) | 29 |
| 13 | One lactation only, breeding stopped, then MC | 5·1 (3·3–7·0) | 6·2 (4·0–9·0) | 9 (6) | 70 |

It will be seen that breast tumours occurred in all the groups of animals. The incidence in the forced breeding group (69 per cent) and in the mice allowed to suckle one litter only before carcinogen treatment (70 per cent) was not significantly different from the incidence obtained in virgins (74 per cent), see Table I. In the natural breeding group given MC before the first lactation the incidence of tumours was lower (29 per cent) than in virgins, but this was not statistically significant ($P = 0{\cdot}1$) and the tumours were not eliminated altogether, as when the MC treatment was begun after the first lactation. In the pseudo-pregnant group, on the other hand, an incidence of 100 per cent was obtained, although the survival of this group was slightly less than that of the others. The difference in proportions between the pseudo-pregnant and virgin mice bearing breast tumours is not statistically significant.

The ovaries of the forced breeding group and those of the pseudo-pregnant group showed some large corpora lutea, primordial follicles, few or no large follicles and often a good deal of blood vessel congestion. The mice which were allowed to lactate once, and were then prevented from breeding further, had ovaries which were diffusely luteinised. Atretic follicles, but few or no developing follicles or corpora lutea were present in them. Some showed blood vessel congestion and one of the 13 had a microscopically detected early granulosa-celled tumour.

Table IV shows the breeding history of the breeding mice in the above experiments. It includes some mice which did not come to autopsy.

From this table it can clearly be seen that, when the carcinogenic treatment is begun at mating, fertility is reduced in both forced breeding and natural breeding groups.

TABLE IV.—*Effect of MC Treatment on Forced Breeding and Natural Breeding IF Mice*

| Mice | Treatment | Number of litters before 1st painting | Mean number of litters after 1st painting (range) |
|---|---|---|---|
| 4 | Forced breeding | 0 | 1·5 (1–2) |
| 9 | ,, ,, | 1 | 3·9 (2–5) |
| 9 | Natural breeding | 0 | 1·6 (1–2) |
| *18 | ,, ,, | 1 | 3·8 (1–7) |

* Included from Table II.

### DISCUSSION

The results of the first two experiments confirm the fact previously reported (Marchant, 1955) that natural breeding with full lactation significantly lowers the incidence of breast tumours induced by chemical carcinogens in IF mice. Although two of the nine breeders treated with DMB developed breast tumours, these occurred in the only two mice of the group which were subsequently found to have ovarian tumours.

The results shown in Table I suggest that DMB is a more potent breast carcinogen than MC, for although the incidence of breast tumours is the same with both carcinogens, the much shorter survival of the DMB-treated group indicates a more rapid induction of breast tumours with this carcinogen. Another fact in support of this is the higher incidence of multiple tumours in virgin mice after treatment with DMB. There was also a higher proportion of tumours in the DMB-treated mice which showed squamous metaplasia.

Tables I and II show that DMB has a much greater effect on ovaries of IF mice than MC, producing a high incidence of granulosa-cell tumours in virgins and lowering the fertility of the breeders. Histological comparisons of the ovaries showed the essential difference was the complete destruction of oocytes by DMB, resulting in a disappearance of follicles.

Methylcholanthrene itself apparently has some effect in reducing fertility. Orr (1946) described the failure of 12 IF females to breed after treatment with MC paintings for 86–149 days. Table IV shows that, when carcinogen treatment is begun before breeding, subsequent fertility is impaired. This table also shows that a single pregnancy before carcinogen treatment has a protective effect against subsequent impairment of fertility.

With regard to the lowering of the incidence of chemically induced breast tumours by natural breeding, it can be seen from the third experiment that repeated lactation is the essential factor. Forced breeding did not result in a lower incidence than in virgins and pseudo-pregnancy actually raised the incidence. A single lactation had no effect in lowering the incidence of breast tumours in mice subsequently treated with MC and prevented from breeding again. This seemed to indicate that repeated lactations during the carcinogen treatment were essential. However, natural breeders given the first carcinogen treatment before breeding produced some breast tumours. To obtain the best protection against breast tumours, it was necessary for the first litter to be suckled before the carcinogen treatment began.

That the protective effects of lactation against the chemical induction of breast tumours do not apply to all strains of mice reputedly free from milk factor, has recently been shown by Ranadive and Hakim (1958). Using the L(P) strain, which had been bred for 8 years in India without producing spontaneous breast tumours, they obtained no breast tumours in virgins treated with MC but a high incidence in natural and forced breeders. It would therefore be of great interest to compare the L(P) and IF strains of mice from an endocrinological point of view.

### SUMMARY

1. A high incidence of breast tumours (77 per cent) was induced by painting with dimethylbenzanthracene (DMB) in virgin mice of the IF strain, which is free from the mammary tumour agent.

2. A lower incidence (22 per cent) was induced in naturally-breeding IF mice which were allowed to lactate before DMB treatment began.

3. No breast tumours were induced in 7 naturally breeding IF mice by the inhalation of methylcholanthrene (MC), though they have previously been obtained in 69 per cent of virgins so treated.

4. The incidence of breast tumours obtained after MC painting was as high in forced-bred females (69 per cent) as in virgins previously reported (74 per cent),

5. One lactation only, followed by MC painting and no further breeding, gave a breast tumour incidence of 70 per cent.

6. Naturally-breeding females in which MC paintings were begun before breeding had an incidence of 29 per cent.

7. Pseudo-pregnancy raised the incidence of breast tumours to 100 per cent.

8. The effects of the various procedures used on the histological structure of the ovary and on fertility is discussed.

9. It is concluded that DMB is more potent in its action on breasts and ovaries than MC.

10. The greatest protection against the induction of breast tumours by DMB and MC in IF mice is afforded by continued pregnancies with full lactation, especially if lactation has commenced before the carcinogen is first applied.

I am grateful to Professor J. W. Orr for allowing me to include data from some of his experiments, and to the Birmingham Branch of the British Empire Cancer Campaign for support of this work.

### REFERENCES

Bonser, G. M.—(1938) *J. Path. Bact.*, **46**, 581.—(1954) *Ibid.*, **68**, 531.
Dmochowski, L. and Orr, J. W.—(1949) *Brit. J. Cancer*, **3**, 520.
Howell, J. S., Marchant, J. and Orr, J. W.—(1954) *Ibid.*, **8**, 635.
Macdonald, I.—(1942) *Surg. Gynec. Obstet.*, **74**, 75.
Marchant, J.—(1955) *J. Path. Bact.*, **70**, 415.—(1957) *Brit. J. Cancer* (in press).
Orr, J. W.—(1943) *J. Path. Bact.*, **55**, 483.—(1946) *Ibid.*, **58**, 589.—(1951) *Acta Union. Internat. contre. Cancer*, **7**, 294.
Ranadive, K. J. and Hakim, S. A.—(1958) *Brit. J. Cancer* **12**, 44.
Versluys, J. J.—(1955) *Ibid.*, **9**, 239.
Wainwright, J. M.—(1931) *Amer. J. Cancer*, **15**, 2610.